# Oral Squamous Cell Carcinoma in Young Patients Show Higher Rates of *EGFR* Amplification: Implications for Novel Personalized Therapy

**DOI:** 10.3389/fonc.2021.750852

**Published:** 2021-11-29

**Authors:** Laveniya Satgunaseelan, Sean Porazinski, Dario Strbenac, Aji Istadi, Cali Willet, Tracy Chew, Rosemarie Sadsad, Carsten E. Palme, Jenny H. Lee, Michael Boyer, Jean Y. H. Yang, Jonathan R. Clark, Marina Pajic, Ruta Gupta

**Affiliations:** ^1^ Department of Tissue Pathology and Diagnostic Oncology, NSW Health Pathology, Royal Prince Alfred Hospital, Sydney, NSW, Australia; ^2^ Sydney Medical School, Faculty of Medicine and Health Sciences, The University of Sydney, Sydney, NSW, Australia; ^3^ Cancer Theme, The Kinghorn Cancer Centre, Garvan Institute of Medical Research, Sydney, NSW, Australia; ^4^ St Vincent’s Clinical School, Faculty of Medicine, University of New South Wales, Sydney, NSW, Australia; ^5^ School of Mathematics and Statistics, The University of Sydney, Sydney, NSW, Australia; ^6^ The Sydney Informatics Hub, Core Research Facilities, The University of Sydney, Sydney, NSW, Australia; ^7^ Sydney Head and Neck Cancer Institute, Department of Head and Neck Surgery, Chris O’Brien Lifehouse, Sydney, NSW, Australia; ^8^ Department of Medical Oncology, Chris O’Brien Lifehouse, Sydney, NSW, Australia; ^9^ Charles Perkins Centre, The University of Sydney, Sydney, NSW, Australia; ^10^ Royal Prince Alfred Institute of Academic Surgery, Sydney Local Health District, Sydney, NSW, Australia

**Keywords:** oral squamous cell carcinoma, *EGFR*, genomics, personalized therapy, tumor mutation burden

## Abstract

There is an increasing worldwide incidence of patients under 50 years of age presenting with oral squamous cell carcinoma (OSCC). The molecular mechanisms driving disease in this emerging cohort remain unclear, limiting impactful treatment options for these patients. To identify common clinically actionable targets in this cohort, we used whole genome and transcriptomic sequencing of OSCC patient samples from 26 individuals under 50 years of age. These molecular profiles were compared with those of OSCC patients over 50 years of age (n=11) available from TCGA. We show for the first time that a molecular signature comprising of *EGFR* amplification and increased *EGFR* RNA abundance is specific to the young subset of OSCC patients. Furthermore, through functional assays using patient tumor-derived cell lines, we reveal that this *EGFR* amplification results in increased activity of the EGFR pathway. Using a panel of clinically relevant EGFR inhibitors we determine that an *EGFR*-amplified patient-derived cell line is responsive to EGFR inhibition, suggesting *EGFR* amplification represents a valid therapeutic target in this subset of OSCC patients. In particular, we demonstrate sensitivity to the second-generation EGFR tyrosine kinase inhibitor afatinib, which offers a new and promising therapeutic avenue versus current EGFR-targeting approaches. We propose that testing for *EGFR* amplification could easily be integrated into current diagnostic workflows and such measures could lead to more personalized treatment approaches and improved outcomes for this younger cohort of OSCC patients.

## Introduction

The advent of next generation sequencing (NGS) has revolutionized our understanding of carcinogenesis and cancer care in the past decade (1). Genetic changes that may lead to carcinogenesis at a younger age or after relatively low exposure to carcinogens are being increasingly identified in a range of malignancies. For example, pulmonary adenocarcinomas in young, non-smoking patients show more frequent *EGFR* (epidermal growth factor receptor) exon 19 deletions and *ALK* (anaplastic lymphoma kinase) fusions, as compared with older patients with lifetime smoking habits (2). Similarly, melanomas in young patients with low cumulative solar ultraviolet light exposure are 2.7 times more likely to show *BRAF* (B-Raf Proto-Oncogene) mutations (3). These changes are amenable to therapeutic targeting which have resulted in improved survival in *ALK*-positive lung cancer and *BRAF*-mutant melanoma (4, 5). These findings suggest that malignancies occurring at a younger age and in the absence of conventional cancer risk factors, may harbor different genetic profiles when compared with older patients. Importantly, these genetic changes may be therapeutically actionable.

Oral cavity squamous cell carcinoma (OSCC) that is independent of human papillomavirus, more commonly occurs in males with a longstanding history of tobacco and alcohol use, at a median age of 61 years (6–8). Over the past decade, several studies have documented the emergence of a new demographic in OSCC, with a two- to four-fold increase in the incidence in patients younger than 45 years of age, despite falling rates of smoking (9–14). Notably, young females account for the majority, increasing at a rate of 4.9% per year (15). A recent review of all head and neck SCC (HNSCC) trials in the United States showed that trial cohorts were largely composed of older men with history of tobacco use, with a significant underrepresentation of young and female patients (16, 17). The clinical trial population in HNSCC is therefore unlikely to be representative of this emerging cohort of young patients with OSCC and therapeutic options therefore need to be explored.

In this study, using whole genome and whole transcriptome sequencing of OSCC tissues from patients younger than 50 years, we characterized the differences in the genomic and transcriptomic profiles with OSCC tissue obtained from patients older than 50 years. The differences in genomic and transcriptomic profiles of OSCC from men and women and smokers and non-smokers were also evaluated. Our data is the first to demonstrate *EGFR* amplification was limited to a subset of young OSCC patients, with concomitant increases in RNA abundance. Functional validation of actionable targets identified through these analyses in patient-derived cell lines revealed a potential therapeutic benefit of EGFR inhibition in *EGFR*-amplified OSCC.

## Materials And Methods

### Patient Selection

After institutional human research ethics committee approval (X19-0282/ETH12165), a search of the Sydney Head and Neck Cancer Institute (SHNCI) databased identified 17 patients younger than 50 years of age with OSCC from 2013-2019. Fresh frozen tissues with optimal DNA, RNA and complete clinicopathologic data were available for all 17 patients. Searching the TCGA database yielded nine patients younger than 50 years of age with OSCC. These patients were included in a validation cohort. The TCGA database also included 11 patients older than 50 years of age with OSCC. These patients were included as a comparison cohort. Complete clinicopathological data including demographic, smoking, histopathology, treatment and survival data were available for the SHNCI cohort. Tumor infiltrating lymphocytes (TILs) were assessed using an established four-tier system (18), with a score of 0 given where there was no inflammatory cells; 1 representing a mild, patchy increase in inflammatory cells; 2 representing a moderate inflammatory infiltrate with some tumor island destruction; and 3 representing a florid inflammatory infiltrate with notable tumor island destruction. Smoking status was dichotomized as ‘never smoker’ (i.e. those with no history of tobacco use) and ‘ever smoker’ (i.e. those with any history of tobacco use). All SHNCI patients were screened with HPV *in situ* hybridization (ISH). One patient demonstrated HPV35 integration, and all other patients were HPV-negative. The available clinicopathologic data for the TCGA validation and comparison cohort were obtained from the Genomic Data Commons Portal (https://portal.gdc.cancer.gov/projects/TCGA-HNSC; dbGaP Study Accession #20551, accessed 7 October 2019). This included information regarding age, gender, smoking and survival status. Histopathologic characteristics of the tumor, treatment or progression-free or overall survival were not available.

### DNA and RNA Extraction

Nucleic acids were extracted from fresh frozen OSCC tissue and matched normal (oral mucosa) using the Qiagen AllPrep Kit (Qiagen, Germantown, Maryland, USA) in accordance with the manufacturer’s instructions with purification of DNA and RNA from the same sample. DNA and RNA were quantified using Qubit V2.0 HS assays (Invitrogen, Carlsbad, USA), NanoDrop spectrophotometry (Thermo Scientific, Waltham, Massachusetts, USA) and 0.8% agarose gel electrophoresis.

DNA integrity was evaluated using the Illumina-CytoSNP-850K array (Illumina, San Diego, California, USA). Sample ploidy and purity were then assessed using “Allele-specific copy number analysis of tumors” (ASCAT) (19), to determine adequate tumor cellularity within samples. Those samples determined by ASCAT to contain an adequate tumor cell fraction (>30%) were submitted for whole genome sequencing (19). RNA quality was assessed using a BioAnalyzer (Agilent Technologies, Wilmington, Delaware USA). Samples with a RNA integrity number >8 were included (20) and only samples with high quality DNA and RNA were included in the study.

### Sequencing

RNA was prepared using the TruSeq Stranded Total RNA with RiboZero kit. Both DNA and RNA sequencing was performed using the Illumina NovaSeq 6000 platform (NovaSeq Xp kit) at the Australian Genome Research Facility (AGRF) on the S4 flow cell to generate 150 base pair (bp) paired-end reads.

Tumoral DNA was sequenced at a target depth of 60X and non-tumoral DNA at 30X. Whole-genome sequencing (WGS) coverage was a median of 85X for tumor tissue (range 64X-140X) and 45X for normal tissue (range 16X-75X) for the SHNCI patients. For the TCGA cohort, WGS had a median coverage of 76X for tumor tissues (range 34X-85X) and 42X for normal tissues (range 30X-52X).

### Short Read Alignment and Short Variant Calling

Raw FASTQ files were mapped to the hg38 reference genome and its alternate contigs with BWA-MEM read aligner (21) to obtain base quality score recalibrated, duplicated marked mapped BAMs. We use GATK v4.1.2.0 tools to call single nucleotide polymorphisms (SNPs) and insertion-deletion (indel) variants. Variants were annotated with their likely effects using ENSEMBL Variant Effect Predictor version 99·2 on default settings except for –pick which selects one effect per variant based on a list of ranked criteria (22). Tumors were assigned an aneuploidy score, which was defined as the total number of altered chromosome arms (23). The workflow is adapted (or based on) BROAD’s best practices workflow for “data pre-processing for variant discovery”, “germline short variant discovery (SNPs + Indels)” and “somatic short variant discovery (SNVs + Indels)”. The detailed description of the methods is available on the Sydney Informatics Hub Github repository (24–26).

Catalogue of Somatic Mutations in Cancer (COSMIC) mutational signature analysis was performed using both COSMIC v2 (27) and COSMIC v3 (28). Both versions yielded similar results for both the SHNCI and the TCGA cohorts. Thus all mutation signature data are presented in the most recent COSMIC v3 format (28), using the MutationalPatterns Bioconductor package (29). Mutational profiles are reconstructed using the relative contributions of known COSMIC v3 signatures in each sample.

### Purity and Ploidy

We inferred the purity and ploidy across each cancer sample’s genome using the AMBER-COBALT-PURPLE pipeline provided in HMF Tools (30, 31). Whole-genome duplication (WGD) is reported as binary value in each sample’s summary file generated by PURPLE (PURity and Ploidy Estimator, version 2·4·1). The value ‘true’ is returned based on whether at least 11 of the 22 autosomes have a major allele ploidy exceeding 1·5 for at least half of the bases in the autosome. Non-integer copy number estimates were rounded to the nearest integer and a copy number of 0 or 1 was considered a deletion and a copy number of 6 or more was considered a high-level amplification.

### Gene-Level Copy Number Changes

Amplifications and deletions are defined based on the TCGA Project’s convention (32). The definition is dependent on whether the cancer sample is inferred to be mostly diploid or having undergone whole genome duplication. If a sample is diploid, then deletion is defined as a gene which has a copy number of zero and amplification is defined as a gene which has a copy number of five or more. However, if a sample is inferred to have had its genome duplicated, a deletion is defined as a gene with a copy number of at least three copies less than the sample ploidy and an amplification is defined as a gene which has at least eight copies.

### Arm-Level Copy Number Changes

A chromosome arm is defined to be deleted if at least half of its bases are one or more copies less than the sample ploidy. A chromosome arm is defined to be amplified if at least half of its bases are one or more copies more than the sample ploidy.

### RNA-Seq Preprocessing

RNA sequencing data was preprocessed using a custom pipeline. Firstly, Illumina TruSeq adapters were trimmed using cutadapt version 2.4 (33) which also removed consecutive bases from 3’ read ends with a Phred-scale quality score of below 20. Next, STAR version 2.7.2 (34) was used to align the reads to the GRCh38 reference genome as well as output alignments in transcriptomic coordinates using the option –quantMode TranscriptomeSAM. The transcript database GENCODE Genes version 31 was used. Reads mapping to multiple locations were allowed and such read alignments were assigned to a particular gene using RSEM (35).

### Fluorescent *In Situ* Hybridization (FISH) Studies

FISH was performed as an orthogonal method to evaluate the copy number calling of the bioinformatics pipeline using a tissue microarray (TMA) constructed from the formalin fixed paraffin embedded SHNCI samples. Interphase FISH for *EGFR* (Vysis *EGFR*/CEP7 Probe Kit, Abbott, Wiesbaden, Germany) was undertaken in a clinically accredited laboratory. Deparaffinization of unstained 4µm formalin-fixed paraffin-embedded tissue sections was performed. Heat-induced epitope retrieval (HIER) pre-treatment with a neutral buffered solution (SPoT-Light Tissue Pretreatment Solution, Thermo Scientific, MA, USA) was then carried out. Tissue sections underwent proteolytic digestion with Protease 1 (Abbott Molecular, IL, USA). A saline-sodium citrate (SSC) buffer rinse then followed. Probe application and denaturing of both the target chromosome and gene probe for 5 minutes at 95°C was completed, after which overnight probe hybridization at 37°C occurred. Dehydration and counterstaining with 4,6-diamidino-phenyldinol (SlowFade™ Gold DAPI, Invitrogen, CA, USA) was subsequently performed. Enumeration of the interphase signals in 100 tumor cell nuclei was undertaken with an epifluorescence microscope (Zeiss, CA, USA).

### Immunohistochemistry (IHC)

Immunohistochemical staining for EGFR was performed to evaluate the protein expression following EGFR CNV using the Leica BOND-III automated staining platform (Leica Biosystems, Melbourne, Australia) according to the manufacturer’s instructions. Bond enzyme pretreatment was undertaken for 15 minutes at 37°C. The primary antibody (EGFR; DAKO; clone: H11, dilution 1:100) was incubated for 30 minutes at ambient room temperature. Leica BOND Polymer Refine Detection was used according to standard BOND-III protocol. Immunohistochemical expression was quantified based on a clinically established four-tier system used to evaluate HER2 IHC expression (36): negative, no staining; 1+: cytoplasmic staining or faint incomplete membrane staining in >10% of tumor cells; 2+: unequivocal complete membrane staining of weak to moderate intensity in >10% of tumor cells; 3+, unequivocal membrane staining of strong intensity in >10% of tumor cells.

### Patient-Derived Cell Line (PDCL) Generation and Culture

OSCC cell lines were generated by mechanically and enzymatically dissociating patient-derived tumors using the human Tumor Dissociation Kit (Miltenyi Biotec, AU) and gentleMACS Octo dissociator in accordance with the manufacturer’s instructions. Cell suspensions were plated onto flasks coated with 0.2mg/ml rat tail I collagen (BD Biosciences, USA) and cultured at 37°C in a 5% CO2 humidified incubator. The TKCC-OSCC-16 and TKCC-OSCC-22 cell lines were cultured in DMEM/F-12 (Thermo Fisher Scientific, AU) supplemented with fetal bovine serum (10%), 15mM HEPES (Thermo Fisher Scientific, AU) and 400ng/mL hydrocortisone (Sigma-Aldrich, USA). For TKCC-OSCC-22 cells, the media was further supplemented with 10ng/mL human recombinant epidermal growth factor (Thermo Fisher Scientific, AU), 0.1IU/mL insulin (Novo Nordisk, DK) and 1x MEM vitamins.

### Western Blotting

Lysates for western blotting were generated by harvesting log-phase cells, washing twice in PBS and lysing at 4°C in lysis buffer [Glycerol (10%), MgCl2 (0.03%), HEPES (1.2%), SAPP (1%), Triton (1%), NaCl (0.8%), NaF (0.4%), EGTA (0.04%)] supplemented with protease inhibitors [MG132 (21µM), Aprotinin (1.5µM), DTT (1µM), Leupeptin (23µM), Sodium Vanadate (1mM), PMSF (1µM)]. Cell debris was removed *via* centrifugation and protein concentration quantified using a Pierce BCA assay kit (Thermo Fisher Scientific, AU). Samples (20µg total protein) were resolved by SDS-PAGE on a Bis-Tris gel (4-12%) followed by transfer to a PVDF (0.45µM) membrane (Life Technologies, AU). Membranes were blocked in 5% non-fat milk in TBS-T [NaCl (0.87%), Tris (0.12%), Tween20 (0.1%)] followed by overnight primary antibody incubation at 4°C. Antibodies for EGFR (#4267) and pEGFR (Tyr1068, #2234) were purchased from Cell Signaling Technologies (Genesearch, AU). Detection was performed using an HRP-conjugated enhanced chemiluminescence-based system (Perkin-Elmer, AU) and relative protein expression was quantified using ImageJ2 Software (V2.1.0, NIH, US).

### *In Vitro* Cytotoxicity Assays

To examine the potential of EGFR as a therapeutic target in the *EGFR*-amplified OSCC, we performed cytotoxicity assays using a panel of six small molecule EGFR inhibitors with differing specificity on our PDCLs and compared these to select publicly available drug sensitivity data (37, 38). Cells for cytotoxicity assays were seeded in 96-well plates and 24h post-seeding, cetuximab (Eli Lilly/Merck), erlotinib, lapatinib, afatinib, gefitinib or saracatinib (all from Selleck Chemicals, USA) were added. Cell viability was measured at 96h post-seeding with alamarBlue (Life Technologies, AU) assays using a FLUOstar Omega (BMG LABTECH, Ortenberg, Germany) plate reader (Excitation 530-560nm, Emission 590nm). IC_50_ values were calculated using GraphPad Prism (V9.0.0, GraphPad, La Jolla, California, USA).

### Statistical Analysis

The median and median absolute deviations (MAD) were calculated for tumour mutation burden (TMB). The differences in the TMB were assessed using Mann Whitney U test. Comparisons of SNVs, CNVs and chromosomal differences were performed using Fisher’s exact test. Statistical comparisons for cytotoxicity assays were performed with unpaired two-tailed *t-tests* using GraphPad Prism. Data are reported as means and error bars show standard error of the mean. Survival differences between groups of patients were compared using a log-rank test.

## Results

### Clinicopathological Characteristics of the OSCC Cohorts <50 Years of Age and ≥ 50 Years of Age

Overall, the study included 37 OSCC patients, with 26 patients under 50 years of age and 11 patients over 50 years of age. The 26 patients <50 years included 17 patients from the SHNCI database and 9 patients from the TCGA cohort. These 26 patients comprised of 17 (65%) males and nine (35%) females with a median age of 42 years (range 19 to 50 years). The 11 patients ≥50 years from the TCGA cohort was comprised of seven (64%) males and four (36%) females with a median age of 62 years (range 52 to 79 years). Smoking data was available for 21/26 patients <50 years. Of these, 11 (52%) were smokers and 10 (48%) were non-smokers. Of the 11 patients ≥ 50 years, there were 10 (91%) smokers and one non-smoker. The clinicopathologic characteristics of the cohort are described in **Table 1**.

**Table 1:** Cohort demographics.

SHNCI cohort < 50 years (n = 17)		
**Age (years) (median, range)**	42 (21-50)	
**Sex:**		***24-month progression-free survival (%)***
Males	10	56
Females	7	86 (log rank test, p=0.21)
**Smoking:**		
Ever smokers	7	50
Never smokers	10	80 (log rank test, p=0.20)
Data not available	0	
**Deceased**	5	
***TCGA cohort < 50 years (n=9)***		
**Age (years) (median, range)**	34 (19-39)	
**Sex:**		***24-month progression-free survival (%)***
Males	7	Data not available
Females	2	Data not available
**Smoking:**		
Ever smokers	4	Data not available
Never smokers	0	Data not available
Data not available	5	
**Deceased**	5	
***TCGA cohort ≥ 50 years (n=11)***		
**Age (years) (median, range)**	62 (52-79)	
**Sex:**		***24-month progression-free survival (%)***
Males	7	Data not available
Females	4	Data not available
**Smoking:**		
Ever smokers	10	Data not available
Never smokers	1	Data not available
Data not available	0	
**Deceased**	5	

Follow up and survival data was available for the 17 SHNCI patients. Of these, six patients had recurrent disease and five patients were deceased. Follow-up time ranged between 6 months and 5 years. Survival data was not available in the TCGA cohort and progression-free survival (PFS) could therefore not be assessed. In the SHNCI cohort, males trended towards lower 24-month PFS compared to females (log rank test, p=0.21, **Table 1**). Also, smokers trended towards lower 24-month PFS as compared to non-smokers (log-rank test, p=0.20, **Table 1**).

### Genomic Analyses of OSCC Reveal Similarities Between the Australian (SHNCI) and TCGA Cohorts of Patients <50 Years of Age

Analyses of the coding regions of all 26 OSCC genomes in patients <50 years revealed 278,193 somatic variants, of which 248,475 (89%) were single-nucleotide variations (SNVs) and the remaining were small insertions and deletions (**Supplementary Tables 1, 2**). Microsatellite instability was assessed as a potential driver of carcinogenesis, particularly in the young cohort. All patients <50 years demonstrated microsatellite stability, and no somatic variants were seen in *POLE* and *MUTYH*.

**Figure 1:**
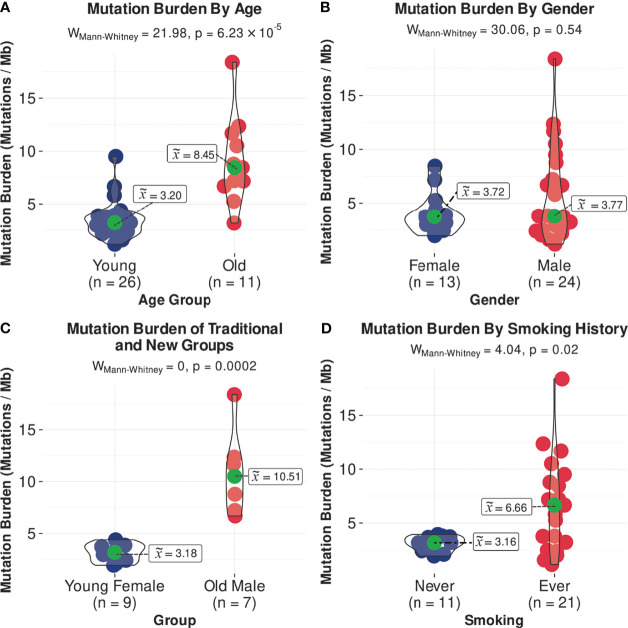
Comparisons of median TMB, **(A)** by age, **(B)** by gender, **(C)** in females <50 years and males >/=50 years, and **(D)** by smoking status.

In the SHNCI cohort, the average TMB was 2.98 variants per megabase (Mb). The TCGA validation cohort of OSCC patients <50 years showed an average TMB of 4.35 variants per Mb. The median TMB of all patients <50 years was 3.20 variants per Mb (MAD = 1.08, **Figure 1A**). A comparison of the median TMB of patients <50 years and those ≥50 years demonstrated a significantly lower TMB in patients <50 years (3.20 variants per Mb, MAD = 1.08) as compared with patients ≥50 years (8.45 variants per Mb, MAD = 3.05) (Mann-Whitney U test, p<0.001, **Figure 1A**; **Table 2**). There was no difference in TMB by sex (**Figure 1B**). Interestingly, the interaction of age and sex yielded a significantly lower median TMB in females <50 years (3.18 variants per Mb, MAD = 1.11), the emerging OSCC demographic, as compared to males ≥50 years (10.51 variants per Mb, MAD = 2.73), the typical OSCC demographic (Mann-Whitney U test, p<0.001, **Figure 1C**; **Table 2**).

**Table 2:** Notable genomic differences by demographic characteristics.

Genomic variant.	Demographic Characteristic.	Variants per Mb .	Mann-Whitney U test p-value.
Tumor mutation burden (TMB)	<50y vs ≥50y	3.20 (MAD = 1.08) vs 8.45 (MAD = 3.05)	<0.001
TMB	Male vs Female	3.77 (MAD = 2.83) vs 3.72 (MAD = 1.11)	0.54
TMB	Female <50y vs Males ≥50y	3.18 (MAD = 1.11) vs 10.51 (MAD = 2.73)	<0.001
TMB	Smokers vs non-smokers	6.66 (MAD = 4.30) vs 3.16 (MAD = 0.83)	0.02
***Genomic variant***	***Demographic Characteristic***	***%***	***Fisher’s Exact Test p-value***
8q arm amplification	< 50y vs ≥ 50y	92% vs 55%	0.02
Chr20 amplification	< 50y vs ≥ 50y	62% vs 9%	0.004
7p11 amplification	< 50y vs ≥ 50y	27% vs 0%	0.08
11q13 amplification	< 50y vs ≥ 50y	82% vs 23%	0.002
*TP53* non-synonymous SNVs	< 50y vs ≥ 50y	85% vs 82%	1
*CDKN2A* non-synonymous SNVs	< 50y vs ≥ 50y	23% vs 36%	1
*LRP1B* non-synonymous SNVs	< 50y vs ≥ 50y	12% vs 45%	0.04
3p arm loss	Male vs Female	92% vs 62%	0.15
7p11 amplification	Male vs Female	25% vs 8%	0.38
11q13 amplification	Male vs Female	42% vs 38%	1
*TP53* non-synonymous SNVs	Male vs Female	88% vs 77%	0.64
*NOTCH1* non-synonymous SNVs	Male vs Female	25% vs 23%	1
7p11 amplification	Smokers vs non-smokers	14% vs 27%	0.39
11q13 amplification	Smokers vs non-smokers	47% vs 27%	0.15
*TP53* non-synonymous SNVs	Smokers vs non-smokers	76% vs 91%	0.64
*NOTCH1* non-synonymous SNVs	Smokers vs non-smokers	29% vs 27%	1

**Figure 2:**
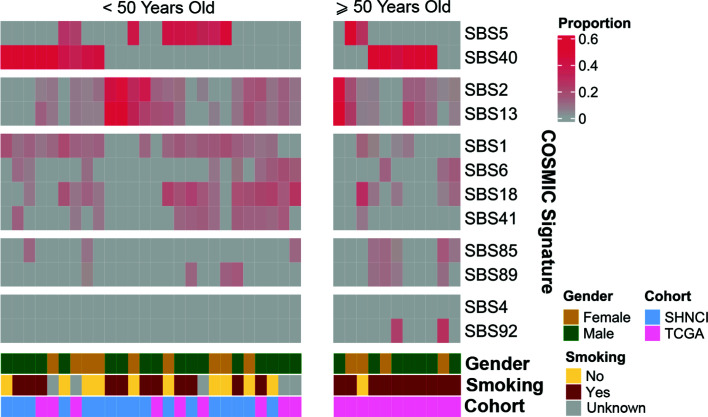
Reconstructed mutational profiles using the relative contributions of known COSMIC v3 signatures in each OSCC sample: COSMIC signature composition for each patient, grouped by age, gender and smoking status.

COSMICv3 single base substitution signature (SBS) 1 and 5 (27, 28), associated with accumulation of mutations with age and characterized by C>T single base substitutions (SBSs) (27, 28), was the dominant pattern. This was seen in 13 (76%) patients from the SHNCI cohort and six patients from the TCGA <50 years cohort (67%, **Figure 2**). No patients, including those with a smoking history demonstrated SBS4 and/or SBS92 signatures, associated with tobacco carcinogens (39) (**Figure 2**).

**Figure 3:**
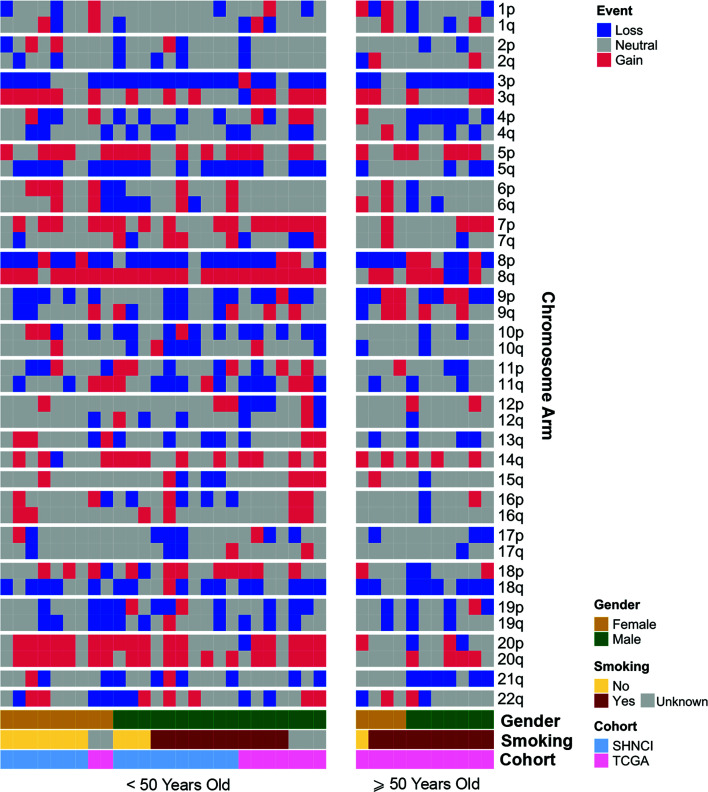
Distribution of chromosomal arm gains and losses in patients <50 years and ≥50 years, grouped by age, gender and smoking status.

The most common chromosomal arm amplifications in the SHNCI cohort included 8q (n=15, 88%), 20q (n=11, 65%) and 20p (n=10, 59%) (**Figure 3**). The most common chromosome arm deletions in the SHNCI cohort included chromosome 3p (40) (n=14, 82%) and chromosome 8p (n=13, 76%). Loss of Y chromosome was seen in three male patients (30% of male patients <50 years). Similar chromosomal arm level amplifications and deletions were seen in the TCGA <50 years cohort, with 8q amplification (n=9, 100%), chromosome 20 amplification (n=7, 79%), 3p deletions (n=7, 79%) and 8p deletions (n=7, 79%) observed (**Figure 3**). RNA sequencing did not demonstrate a resultant increased fold change in transcription factor target genes on 8q, 20q and 20p in either cohort.

**Figure 4:**
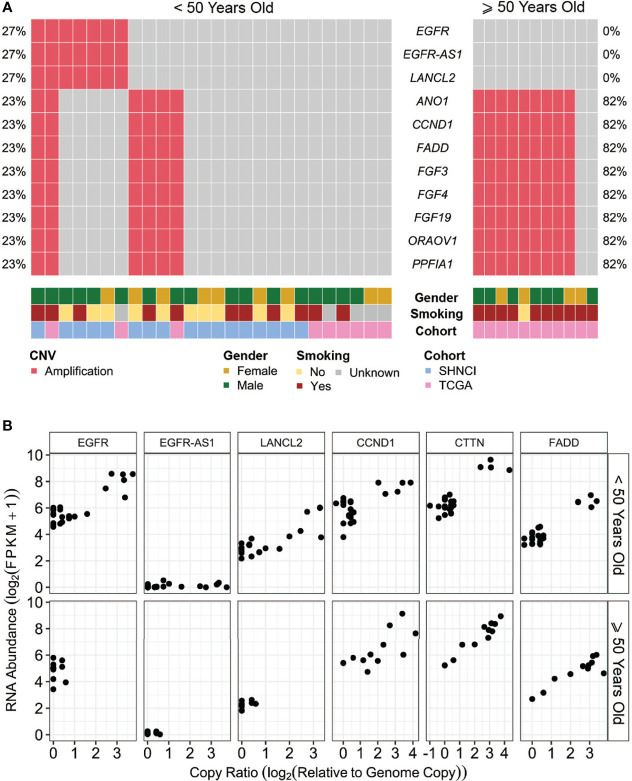
**(A)** Somatic copy number alterations (SCNAs) in patients <50 years and ≥50 years, grouped by age, gender and smoking status: The most common copy number variants occurring in over 20% of patients, with **(B)** associated RNA abundance.

Somatic copy number alterations (SNCAs), including amplifications of *EGFR*, *EGFR-AS1*, and *LANCL2* (located at Chr7p.11, n=5, 29%) and amplifications of *ANO1*, *CCND1*, *FGF3*, *FGF4*, *ORAOV1* and *CTTN* (located at Chr11q.13, n=4, 24%) were present in the SHNCI cohort (**Figure 4A**; **Supplementary Table 3**). Both *EGFR* and *LANCL2* showed increased RNA abundance with increasing relative copy number (**Figure 4B**). In contrast, accompanying changes in RNA abundance were not observed for *EGFR-AS1* or the genes present at 11q.13 (**Figure 4B**). The CNV profile was reproduced in the TCGA <50 years cohort, with amplification at 7p.11 (n=2, 22%) and 11q.13 (n=1, 11%, **Figure 4A**), with a similar transcriptomic profile.

The most common deletion was *CDKN2A/B*, observed in three patients in the SHNCI cohort (18%) and one in the TCGA <50 years cohort (11%). Of these, two patients showed low levels of *CDKN2A* RNA expression, while one patient showed an approximately 10-fold gene abundance increase in RNA. This may be due to an epigenomic phenomenon, such as aberrant methylation or transcription factor binding to the promoter region.

**Figure 5:**
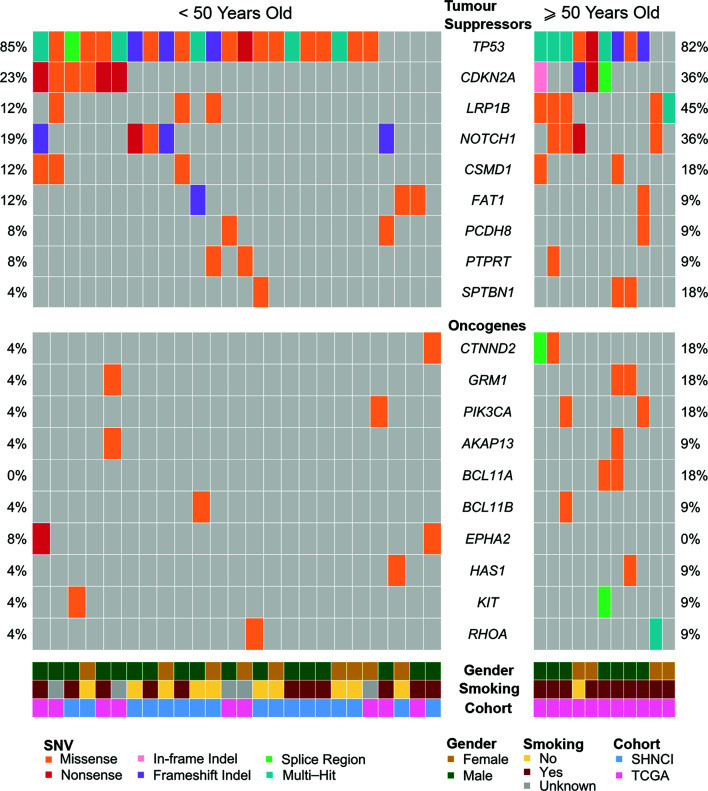
Frequent single nucleotide variants in patients <50 years and ≥50 years, occurring in tumor suppressor genes, and oncogenes.

Non-synonymous SNVs were more frequent in tumor suppressor genes than oncogenes (**Figure 5**). The most common non-synonymous SNVs in the SHNCI cohort were in *TP53* (n=15, 88%), of which 10 patients showed missense mutations with concomitant changes in RNA expression (40). RNA expression could not be evaluated in the remaining five patients who harbored insertion/deletions in *TP53*, due to the potential confounding factor of nonsense mediated decay. The TCGA <50 years cohort demonstrated comparable findings, with the most commonly affected gene being *TP53* (n=7, 78%); six patients (67%) with *TP53* SNVs identified had accompanying changes in RNA expression. The next most commonly affected gene was *CDKN2A*, where SNVs were seen in two patients in the SHNCI group (12%) and four patients in the TCGA <50 years cohort (44%). However, no transcriptomic alterations were detected. Non-synonymous SNVs in oncogenes were rare, with isolated patients showing nucleotide changes in *PIK3CA* (TCGA cohort: n=1, 11%) and *KIT* (SHNCI cohort: n=1, 6%). No RNA changes were apparent as a result of SNVs in the limited oncogenes affected.

FFPE material for histological evaluation of TILs was only available for the SHNCI cohort and showed that the majority of both *EGFR-*amplified and non-amplified cases showed low levels of TILs (**Supplementary Figure 1**).

### The Genomic and Transcriptomic Landscape of OSCC Patients <50 Years and Those ≥50 Years Is Significantly Divergent

There were significant differences in genomic profiles according to age. The median TMB in patients ≥50 years was more than double patients <50 years, with a median TMB of 8.45 (MAD = 3.05) versus 3.20 variants per Mb (MAD = 1.08), respectively (Mann-Whitney U test, p<0.001, **Figure 1A**; **Table 2**). The median TMB for males ≥50 years (10.51 variants per Mb, MAD = 2.73) was more than triple females <50 years (3.18 variants per Mb, MAD = 1.11; Mann-Whitney U test, p<0.001, **Figure 1C**; **Table 2**).

COSMIC SBS2 and SBS13, associated with the activation-induced deaminase (AID) and apolipoprotein B mRNA editing catalytic polypeptide-like (APOBEC) family of cytidine deaminases (27, 28), was the dominant pattern seen in 9/11 (82%) TCGA patients ≥50 years (**Figure 2**).

At the chromosomal arm level, 8q amplification was significantly more common in the patients <50 years [24/26 (92%) versus 6/11 (55%) ≥50 years, Fisher’s exact test, p=0.02, **Table 2**]. Whole chromosome 20 amplification was observed more frequently in patients <50 years (16/26 (62%) versus 1/11 (9%) ≥50 years, (Fisher’s exact test, p=0.004, **Table 2**). All male patients ≥50 years showed Y chromosome deletion, compared with only 3/17 (17%) male patients <50 years (**Figure 3**). Transcriptomic changes were not observed in association with the frequently recurrent chromosome arm level changes.

Interestingly, amplification of *EGFR*, *EGFR-AS1* and *LANCL2* were not seen in any of the patients ≥50 years, versus 7/26 (27%) patients <50 years (Fisher’s exact test, p=0.08, **Figure 4A**; **Table 2**). Conversely, CNVs of 11q.13 with amplification of *CCND1, CTTN, FADD, FGF3* and *FGF4* were more frequent in patients ≥50 years (9/11 (82%) versus 6/26 (23%) patients <50 years; Fisher’s exact test, p=0.002, **Figure 4A**; **Table 2**). Like patients <50 years, an increase in RNA abundance with increase in relative copy numbers of *CCND1*, *CTTN* and *FADD* was observed in patients ≥50 years (**Figure 4B**). An increase in RNA abundance with increase in relative copy numbers was limited to three (27%) patients ≥50 years for *FGF3* and two (19%) patients ≥50 years for *FGF4.*

All patients ≥50 years demonstrated microsatellite stability. While there were no *MUTYH* variants, one patient ≥50 years (11%) harbored a missense mutation in *POLE*. A high frequency of *TP53* variants (nine patients, 82%) was also observed in the ≥50 year cohort (**Figure 5**), of which six patients (55%) showed changes in RNA. SNVs in *LRP1B* occurred in five patients in the ≥50 year cohort (45%), as compared to three patients (12%) in the <50 year cohort. *CDKN2A* mutations occurred at similar frequencies in both cohorts (6/26 (23%) <50 years versus 4/11 (36%) ≥50 years, **Figure 5**).

### Comparison of Genomic and Transcriptomic Characteristics of Male and Female Patients With OSCC

The median TMB in females (3.72 variants per Mb, MAD = 1.11) was similar to males (3.77 variants per Mb, MAD = 2.83; **Figure 1B**), although a significant difference was observed on stratification by age (**Figure 1C**; **Table 2**). There were no differences in COSMIC mutation signature patterns between males and females (**Figure 2**). Chromosomal arm alterations also occurred at similar frequencies, with the exceptions of chromosome 3p loss [eight (62%) females versus 22 (92%) males, **Figure 3**; **Table 2**]. SCNAs were seen predominantly in males. *EGFR*, *EGFR-AS1* and *LANCL2* amplification with increased RNA abundance was found in seven patients, six of whom were male (**Figures 4A, B**; **Table 2**). Focal amplification of chromosome 11q.13, including *CCND1, FGF3 and FGF4* was seen in 13 patients (35%) of which eight were male (**Figure 4A**; **Table 2**). Associated transcriptomic changes were seen in *CCND1*, *CTTN* and *FADD*. Similarly, SNVs in tumor suppressor genes were observed largely in males. SNVs in *TP53* were the most frequent in the cohort, seen in 31 patients overall, with 21 of these being male (**Table 2**). The next most frequent SNV was *NOTCH1* seen in nine patients overall, of which six were male (**Table 2**). As SNVs in oncogenes were uncommon, a sex preponderance was not detected (**Figure 5**).

### Comparison of Genomic and Transcriptomic Characteristics of Smokers Versus Non-Smokers

Overall, there were 11 non-smokers, 21 smokers and smoking information unavailable for five patients <50 years from the TCGA validation cohort. Smokers demonstrated a two-fold increase in TMB (6.66 variants per Mb, MAD = 4.30) compared with non-smokers (3.16 variants per Mb, MAD = 0.83; Mann-Whitney U test, p=0.02, **Figure 1D**; **Table 2**). Non-smokers predominantly showed SBS1 (10/11, 91%), whereas the AID/APOBEC mutational signatures (SBS2 and SBS13) was the most frequent signature in smokers (16/21, 76%). Of note, the tobacco associated signature SBS4 was not seen in any patient and SBS92 was seen in only two smokers (10%) (**Figure 2**).

Smokers were more likely to have 11q.13 (harboring *ANO1*, *CCND1*, *FGF3*, *FGF4*, *ORAOV1* and *CTTN*) amplifications (12/21 (47%) smokers versus 3/11 (27%) non-smokers, Fisher’s exact test, p=0.15; **Figure 4A**; **Table 2**), with concomitant increases in RNA abundance in *CCND1, CTTN and FADD*. Focal amplification of 7p.11 (including the genes *EGFR, EGFR-AS1 and LANCL2*) occurred in three smokers (14%) and three non-smokers (27%) (**Figure 4A**; **Table 2**), with an associated increase in RNA abundance of *EGFR* and *LANCL2* (**Figure 4B**). The SNV profile was also similar, with *TP53* mutations frequent in both smokers (16/21, 76%) and non-smokers (10/11, 91%) (**Table 2**). *NOTCH1* mutations were infrequent in both cohorts (24% in smokers and 27% in non-smokers, **Figure 5**; **Table 2**). All patients for whom smoking data was available were microsatellite stable.

### Validation of the EGFR Amplification Molecular Signature as a Potential Therapeutic Target in OSCC Patients <50 Years

**Figure 6:**
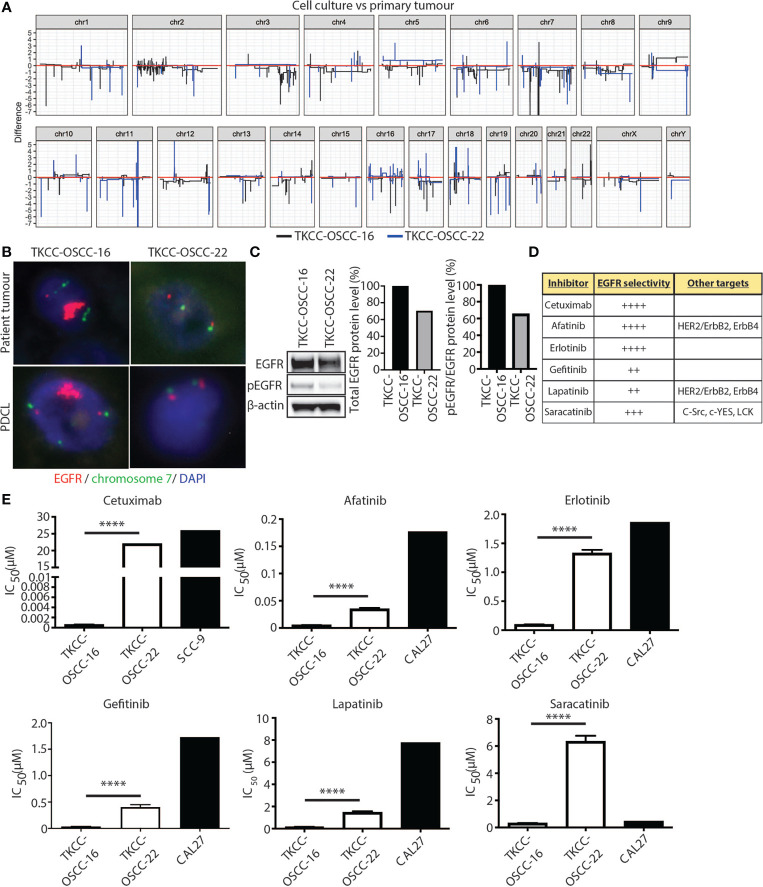
EGFR as a therapeutic target in OSCC patients <50 years. **(A)** Linearized plots of the copy number difference between matched patient tumor samples and PDCLs. **(B)** EGFR FISH for matched patient tumor tissue samples and PDCLs (red = *EGFR* probe; green = chromosome 7 centromeric probe; blue = DAPI). **(C)** Western blotting analysis for total EGFR and pEGFR protein levels in indicated PDCLs. β-actin was used as a loading control. Quantification of protein levels is shown in graphs on right. **(D)** Panel of EGFR inhibitors used in cytotoxicity assays with EGFR selectivity indicated. **(E)** alamarBlue proliferation assays were performed with indicated EGFR inhibitors and IC_50_ values calculated for PDCLs. IC_50_ values for PDCLs generated by us were compared against IC_50_ values for a reference OSCC cell line available from public databases. Significance levels are indicated by asterisks where *P < 0.05, **P < 0.01, ***P < 0.001 and ****P < 0.0001, unpaired t-test.

*EGFR* amplification was observed in 7/26 (27%) OSCCs from patients <50 years with an average of >20 copies per tumor (**Figure 6A**). This represented the only genetic change with currently FDA-approved therapy. Orthogonal confirmation with FISH and IHC on FFPE was only possible for the patients in SHNCI cohort. All patients with *EGFR* CNVs were found to have *EGFR* amplification by FISH (median copy number 15, range 10 to 200, **Figure 6B**). Two cases demonstrated intratumoral heterogeneity with aggregates of markedly *EGFR*-amplified cells surrounded by clusters of SCC cells with similar histologic appearance but no amplification (**Supplementary Figure 2**). Of the five patient tumors with identified *EGFR* amplification, all showed membranous EGFR protein expression (three showed 3+ staining and two showed 2+ staining in >10% of tumor cells; **Supplementary Figure 3**). Patient tumors without *EGFR* SCNAs did not demonstrate *EGFR* amplification by FISH or 3+ membranous staining with IHC. All of the non-amplified cases showed EGFR immunostaining intensity of 0, 1+ and 2+.

To assess the efficacy of EGFR-targeted therapies in *EGFR*-amplified OSCC, we generated two patient tumor-derived primary cell lines (PDCLs), one harboring EGFR-amplification (TKCC-OSCC-16) and one without EGFR-amplification (TKCC-OSCC-22). The TKCC-OSCC-16 line was derived from a primary tongue tumor specimen from a 41-year-old male and the TKCC-OSCC-22 line was obtained from a primary tongue tumor specimen from a 37-year-old male. Both tumor samples and PDCLs underwent WGS and transcriptomic profiling and demonstrated similar genomic (**Supplementary Figures 4**, **5**) and transcriptomic landscapes. Importantly, EGFR copy numbers were concordant between respective tissue and PDCLs (**Figure 6A**). This was confirmed with EGFR FISH analysis of both PDCLs, which showed conservation of *EGFR* CNVs between patient tumor tissue and matched cell lines (**Figure 6B**). Western blot analysis revealed that EGFR protein levels were approximately 30% higher in the EGFR-amplified TKCC-OSCC-16 PDCL versus non-EGFR amplified TKCC-OSCC-22 cells (**Figure 6C**). In line with previous reports (37), we observed low basal levels of EGFR phosphorylation (pEGFR, Tyr1068) in the TKCC-OSCC-22 tumor culture, whereas pEGFR protein levels were 55% higher in the TKCC-OSCC-16 model, suggesting activation of this signaling axis in the *EGFR*-amplified line (**Figure 6C**).

The EGFR-amplified TKCC-OSCC-16 cell line displayed approximately 10-fold increased sensitivity to all inhibitors compared to the TKCC-OSCC-22 cells (**Figure 6D**), except for cetuximab where the difference was much greater (**Table 3**). For afatinib, a dual HER2/neu and EGFR inhibitor, we observed an IC_50_ value of 0.0051µM for TKCC-OSCC-16 and 0.035µM for TKCC-OSCC-22 (**Figure 6E**) suggesting both cell lines were sensitive to this inhibitor. Cetuximab treatment resulted in IC_50_ values of 0.00058µM and 22µM for the TKCC-OSCC-16 and TKCC-OSCC-22 cell lines, respectively, revealing that the cell line harboring the *EGFR* amplification was highly sensitive to cetuximab (**Figure 6E**). Treatment with erlotinib resulted in IC_50_ values of 0.096µM and 1.33µM for TKCC-OSCC-16 and TKCC-OSCC-22, respectively (**Figure 6E**), demonstrating that TKCC-OSCC-22 was resistant to erlotinib. IC_50_ values for gefitinib were 0.034µM and 0.4µM for TKCC-OSCC-16 and TKCC-OSCC-22, respectively (**Figure 6E**), suggesting both cell lines were susceptible to gefitinib. Examination of the dual HER2/neu and EGFR inhibitor, lapatinib, revealed IC_50_ values of 0.16µM for TKCC-OSCC-16 and 1.47µM for TKCC-OSCC-22 (**Figure 6E**), demonstrating TKCC-OSCC-22 was resistant to lapatinib. Finally, saracatinib produced IC_50_ values of 0.32µM for TKCC-OSCC-16 and 6.34µM for TKCC-OSCC-22 (**Figure 6E**), indicating the TKCC-OSCC-22 cell line was highly resistant to saracatinib. In summary, these data demonstrate that the EGFR-amplified TKCC-OSCC-16 cell line was sensitive to all six EGFR inhibitors, with exquisite sensitivity observed for the clinically relevant EGFR inhibitors, cetuximab, afatinib and erlotinib.

**Table 3:** IC_50_ values for cytotoxicity screening.

Drug	IC_50_ value (µM)		p-value
	TKCC-OSCC-16	TKCC-OSCC-22	
Cetuximab	0.00058	22	<0.0001
Afatinib	0.0051	0.035	<0.0001
Erlotinib	0.096	1.33	<0.0001
Gefitinib	0.034	0.4	<0.0001
Lapatinib	0.16	1.47	<0.0001
Saracatinib	0.32	6.34	<0.0001

## Discussion

HPV-independent OSCC has been on the rise in patients <50 years over the past decade, particularly in females and non-smokers (6, 15). Young patients and females are significantly under-represented in clinical trials evaluating therapeutic options in OSCC (16, 17). Indeed, studies of adjuvant treatment in <50 year OSCC patients are often heterogeneous, and while standard treatment protocols of surgery and radiotherapy are well established, targeted therapy protocols are yet to be defined (41). It is increasingly being recognized that in a variety of solid tumors, younger patients with lower cumulative exposure to carcinogens harbor different genomic characteristics, compared to older patients with a lifetime exposure to environmental or lifestyle mutagens (42–44). Our data demonstrate a significantly divergent genomic and transcriptomic landscape in OSCC from patients <50 years and those ≥50 years. For example, young non-smoking females demonstrate significantly lower median TMB (3.18 variants per Mb) as compared with older males with smoking history (10.51 variants per Mb). Co-amplification of *EGFR* and *LANCL2* was seen in younger patients in our study. Amplification of these co-located genes at 7p11 has also been described in glioblastoma, with *LANCL2* amplification portending a poor prognosis in younger glioblastoma patients (45). We also observed *EGFR* amplification with concomitant increase in RNA abundance to be more common in OSCC patients <50 years. We have further demonstrated the clinical utility of this finding by showing high response rates to EGFR inhibitors in a patient-derived cell line with *EGFR* amplification and increased levels of pEGFR protein suggesting activation of the EGFR signaling axis.

As part of this study, we generated two robust 2-dimensional patient-derived cell line models from patients <50 years, one with and one without demonstrable *EGFR* amplification confirmed computationally and by orthogonal methods such as FISH and IHC. The *EGFR*-amplified model, TKCC-OSCC-16 retained *EGFR*-amplification, exhibiting increased *EGFR* activation, compared against the non-amplified model, representing a valuable preclinical tool for the examination of therapeutic efficacy of EGFR-targeting. Importantly, we demonstrate, for the first time, potent efficacy of single agent EGFR inhibitor therapy in a subtype of OSCC with *EGFR* amplification. Broad efficacy was observed across EGFR monoclonal antibody (mAb) cetuximab and five tyrosine kinase inhibitors with differing selectivity for EGFR (plus other kinases, HER2/neu, SRC family), with the most promising signal of activity observed for clinically relevant and selective EGFR inhibitors, cetuximab, afatinib and erlotinib.

Anti-EGFR therapies, chiefly cetuximab, have been studied in HNSCC with modest benefits and variable results (46–48). This may be attributed to the advanced patient age in most HNSCC clinical trials (16, 49), a cohort unlikely to have *EGFR* CNV. Similar to our study, Costa et al (50) and Vincent-Chong et al (51) also demonstrated higher rates of *EGFR* CNV in young patients. Furthermore, most HNSCC clinical trials evaluating anti-EGFR therapies were performed in unselected populations where *EGFR* CNV or EGFR overexpression using immunohistochemistry was not investigated (49). Studies that have evaluated *EGFR* amplification by *in situ* hybridization techniques have demonstrated benefits with either EGFR tyrosine kinase inhibitors (TKIs) or anti-EGFR mAb in non-small cell lung cancer (NSCLC) (52, 53). Thus, the low response rates to EGFR inhibitors in HNSCC trials may not be applicable to younger OSCC patients who have a high rate of *EGFR* amplification. Huang et al. in their proteomics study of HPV-independent HNSCC suggested that elevated protein levels of EGFR may play a role in response to EGFR mAb such as cetuximab (54). These findings imply that both *EGFR* amplification and elevated EGFR protein levels may be used as a biomarker to select patients likely to respond to EGFR inhibitors. Kirchner et al. (55) and Matsumoto et al. (56) have described a ‘cold’ immunophenotype in *EGFR*-mutant NSCLC. Our analysis demonstrates that young patients with *OSCC* appear to have low levels of TILs irrespective of *EGFR* amplification status.

*EGFR* amplification is associated with downstream EGFR protein activation and may potentially present a robust stratifying biomarker for EGFR-targeting therapy. Interestingly, 3+ membranous staining on EGFR IHC entirely correlated with *EGFR* amplification, while the cases with intratumoral heterogeneity demonstrated a 2+ staining pattern. Thus, IHC expression of 2+ and 3+ can be useful in triaging patients for more comprehensive testing of *EGFR* CNV by ISH or polymerase chain reaction as is the current practice for detection of HER2 amplification in breast and gastric cancer (36, 57). Testing for *EGFR* amplification can be easily integrated into the current pathology diagnostic workflow with the use of IHC and FISH, providing results in a cost-effective and clinically appropriate timeframe. However, this will require standardisation of criteria, particularly for copy numbers of the *EGFR* gene and the proportion of tumor cells demonstrating *EGFR* amplification as has been achieved for HER2 in breast (36) and gastric (57) cancer. This is highlighted by our observations of intratumoral heterogeneity for *EGFR* amplification in two patients <50 years. Both tumors showed a subpopulation of tumor cells (approximately 40-50% of tumor cells) with copy numbers >30, with other tumor cells showing normal ploidy. Similar heterogeneity has been described for *HER2* in both breast and gastric cancer and ISH testing of tissues with 2+ and 3+ membranous staining by IHC is recommended for *HER2* testing in gastric cancer (36, 57).

Afatinib represents an important unexplored therapeutic avenue since patients with recurrent and metastatic HNSCC, including OSCC, frequently develop resistance to cetuximab (58–60). *EGFR* mutations are uncommon in HNSCC (61, 62). By contrast, marked upregulation of EGFR *via* amplification or activation *via* one of its ligands (e.g. transforming growth factor alpha, TGF-α) is common (62, 63). Cetuximab is a monoclonal antibody that prevents dimerization of EGFR by binding to its extracellular domain, inhibiting receptor-ligand interactions (61, 64), whereas afatinib is a second-generation TKI that irreversibly binds the intracellular tyrosine kinase domain of EGFR (61, 65). Afatinib has been shown to be more efficacious than chemotherapy for the treatment of NSCLCs harboring specific *EGFR* mutations (55, 56, 66). However, data on the use of afatinib for the treatment of HNSCC is still emerging, although in clinical trials, afatinib has demonstrated higher activity versus other EGFR TKIs (67). Early phase clinical trials suggested similar abilities of afatinib and cetuximab to control disease progression in platinum-resistant HNSCC (68). Furthermore, afatinib has also shown promise as a second-line monotherapy treatment for recurrent/metastatic HNSCC (68, 69). Combination treatments utilising targeted inhibition of EGFR may further improve therapeutic response. EGFR inhibition using gefitinib in combination with cisplatin, has been shown to enhance the pro-apoptotic and anti-proliferative effects of chemotherapy *in vitro* in selected (non-*EGFR* amplified) HNSCC commercially available cell lines (37). Furthermore, combinations of afatinib with cisplatin (70) or cisplatin treatment followed by afatinib (67) in EGFR wild-type HNSCC cells have led to increased cytotoxicity, suggesting such approaches warrant further investigation in the context of *EGFR*-amplified OSCC.

One of the limitations of our study is its sample size. HPV-independent OSCC in those under 50 years, although increasing in incidence, is a rare cancer with devastating consequences for young patients (15). TCGA data was therefore accessed in an effort to increase the sample size, however, only nine cases of HPV independent OSCC <50 years, with whole genome sequencing data, were identified from this international, multi-institutional cohort, indicating the rarity of this cancer. TCGA data also does not provide treatment and survival details for its cohort. However, the comprehensive functional analyses included in the current study highlight the robust nature of our findings.

Our study is the first to identify lower TMB and *EGFR* amplification with associated increased RNA abundance in cases of OSCC <50 years – a much needed advance towards personalized therapy, where the literature to date has recognized limited differences in OSCC patients <50 and ≥50 years (71–73). The genomic, transcriptomic and functional findings of this study provide evidence for testing *EGFR* CNV and pEGFR levels when designing future clinical trials utilizing EGFR targeted therapies. With *EGFR* amplification testing readily available in the clinical diagnostic setting, *EGFR* CNV testing needs to be considered in OSCC patients <50 years of age.

## Data Availability Statement

The original contributions presented in the study are publicly available. This data can be found here: National Center for Biotechnology Information (NCBI) Gene Expression Omnibus (GEO) with accession ID GSE184616 (https://www.ncbi.nlm.nih.gov/geo/query/acc.cgi?acc=GSE184616).

## Ethics Statement

The studies involving human participants were reviewed and approved by Sydney Local Health District, NSW Health, Human research ethics committee approval X19-0282/ETH12165. The patients/participants provided their written informed consent to participate in this study.

## Author Contributions

MP, RG, SP, and LS devised the project and the main conceptual ideas. LS, JC, RG, and CP were responsible for tissue collection. LS collected all included clinicopathological data for the SHNCI and TCGA cohorts. CW, TC, and RS performed the pre-processing of the raw sequencing data. DS and JY performed the computational analysis. FISH study interpretation was performed by LS. LS and RG translated the computational data to the clinicopathological context. SP, AI, and MP performed all patient derived cell line generation and culture, Western blotting and *in vitro* cytotoxicity assays, as well as all related analysis. JC, JL, and MB contributed to the interpretation of the results. LS and SP prepared the manuscript. All authors provided critical feedback and helped shape the analysis and the manuscript. All authors contributed to the article and approved the submitted version.

## Funding

This work was supported by the National Health and Medical Research Council (NHMRC), Australia (Project Grant 2003310 and Fellowship 1162556 to Associate Professor Marina Pajic). This work was also supported by the Australian and New Zealand Head and Neck Cancer Society (ANZHNCS). SP is supported in part by a Tour de Cure mid-career researcher grant (RSP-359-2020).

## Conflict of Interest

The authors declare that the research was conducted in the absence of any commercial or financial relationships that could be construed as a potential conflict of interest.

## Publisher’s Note

All claims expressed in this article are solely those of the authors and do not necessarily represent those of their affiliated organizations, or those of the publisher, the editors and the reviewers. Any product that may be evaluated in this article, or claim that may be made by its manufacturer, is not guaranteed or endorsed by the publisher.
