# Supplementary figures and images for: Oral Squamous Cell Carcinoma in Young Patients Show Higher Rates of EGFR Amplification: Implications for Novel Personalized Therapy
Source: Front Oncol. 2021 Nov 29;11:750852. doi: 10.3389/fonc.2021.750852 (PMC8666981; doi:10.3389/fonc.2021.750852)

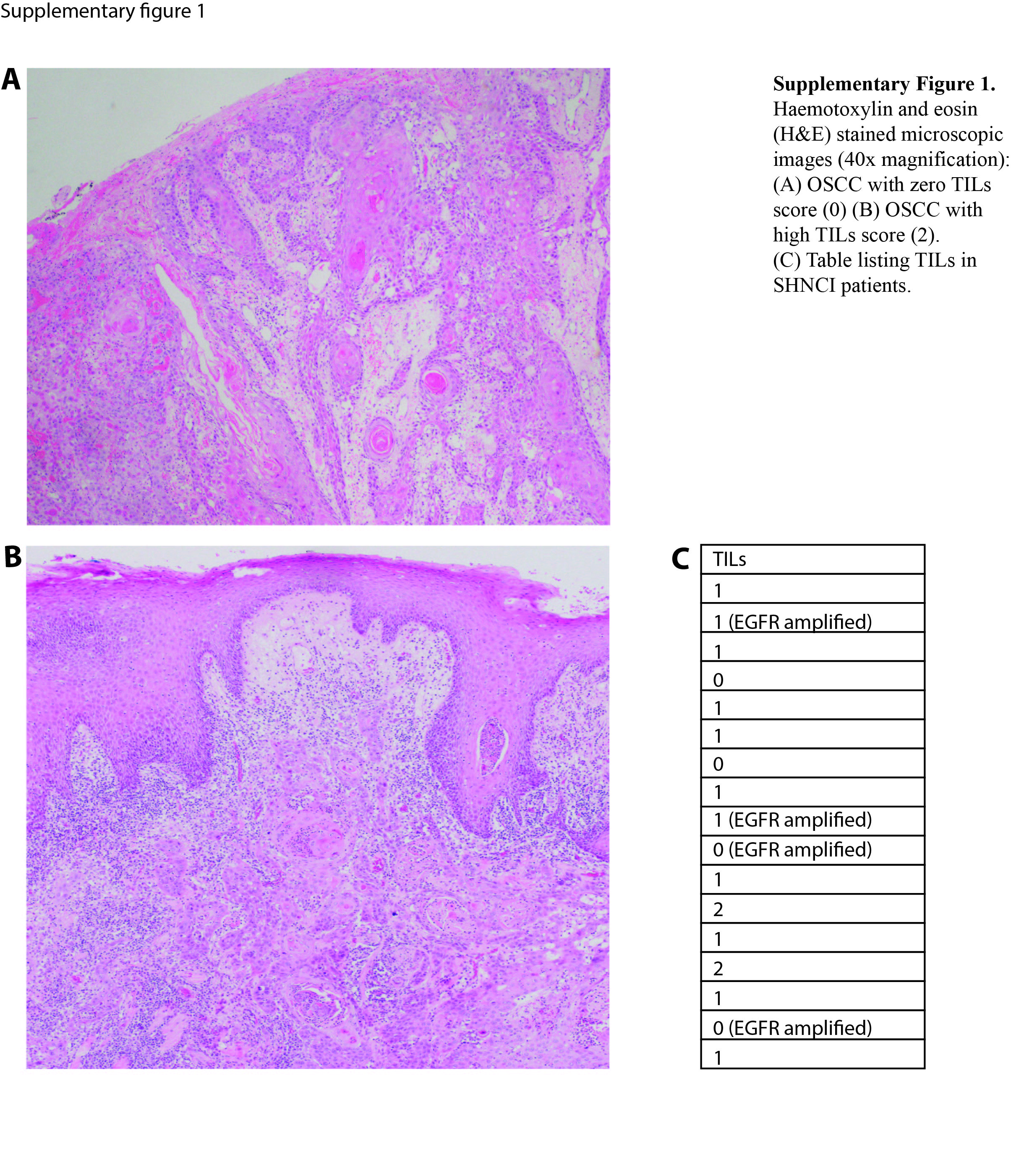

Supplement: Supplementary file 1 [file Image_1.jpeg]

Patient tumour

PDCL

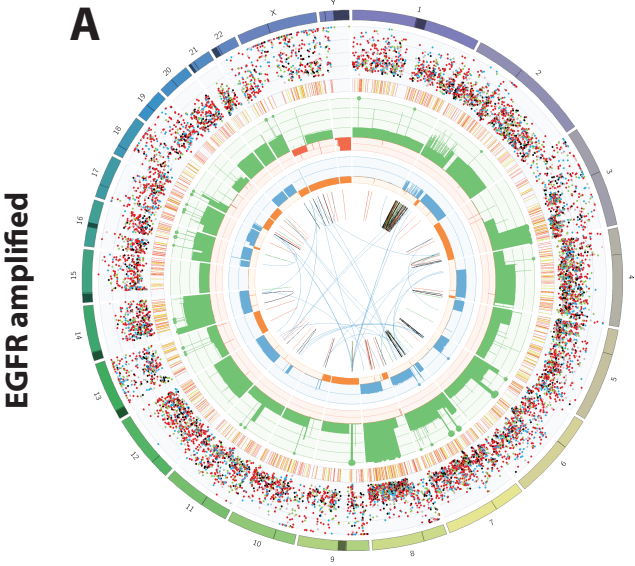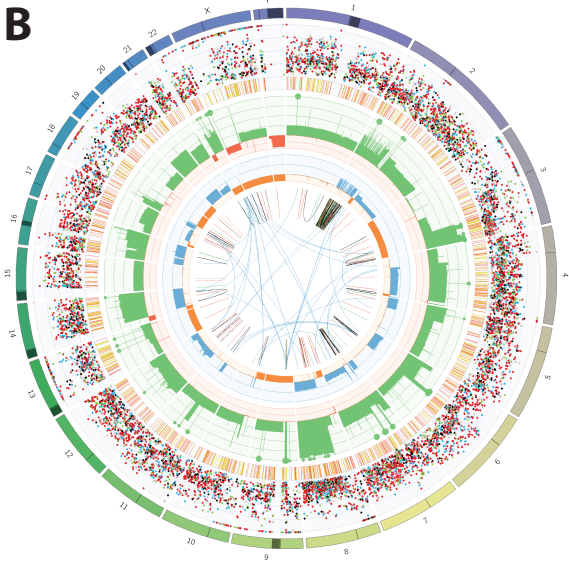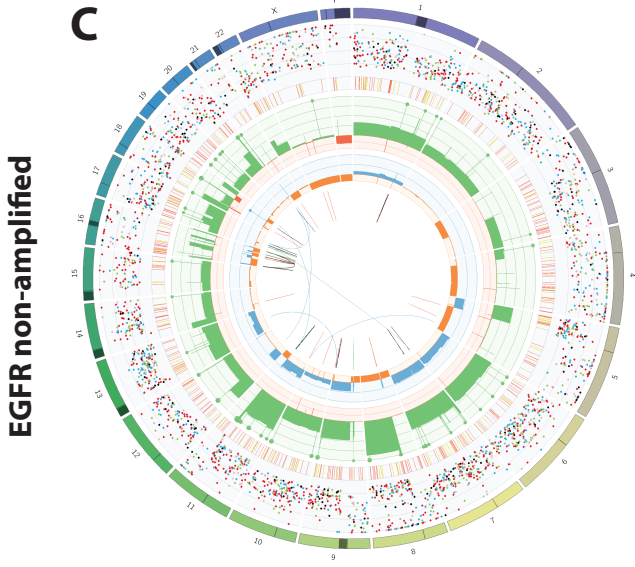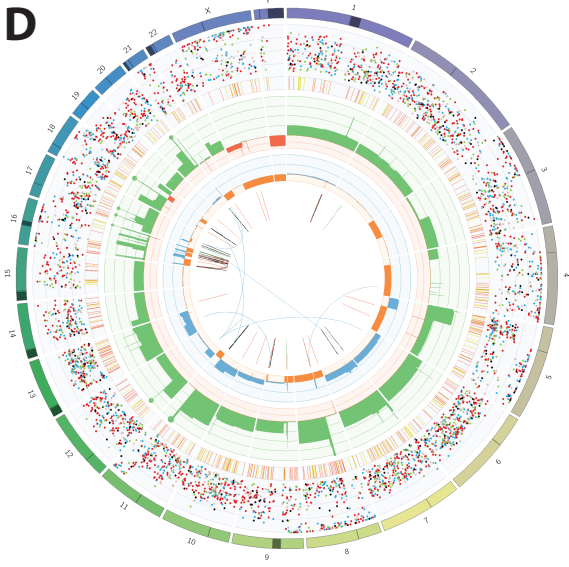

Supplement: Supplementary file 4 [file Image_4.pdf]
