# Supplementary material for: Oral Squamous Cell Carcinoma in Young Patients Show Higher Rates of EGFR Amplification: Implications for Novel Personalized Therapy
Source: Front Oncol. 2021 Nov 29;11:750852. doi: 10.3389/fonc.2021.750852 (PMC8666981; doi:10.3389/fonc.2021.750852)
Supplement: Supplementary file 2 [file Image_2.pdf]

Supplementary figure 2

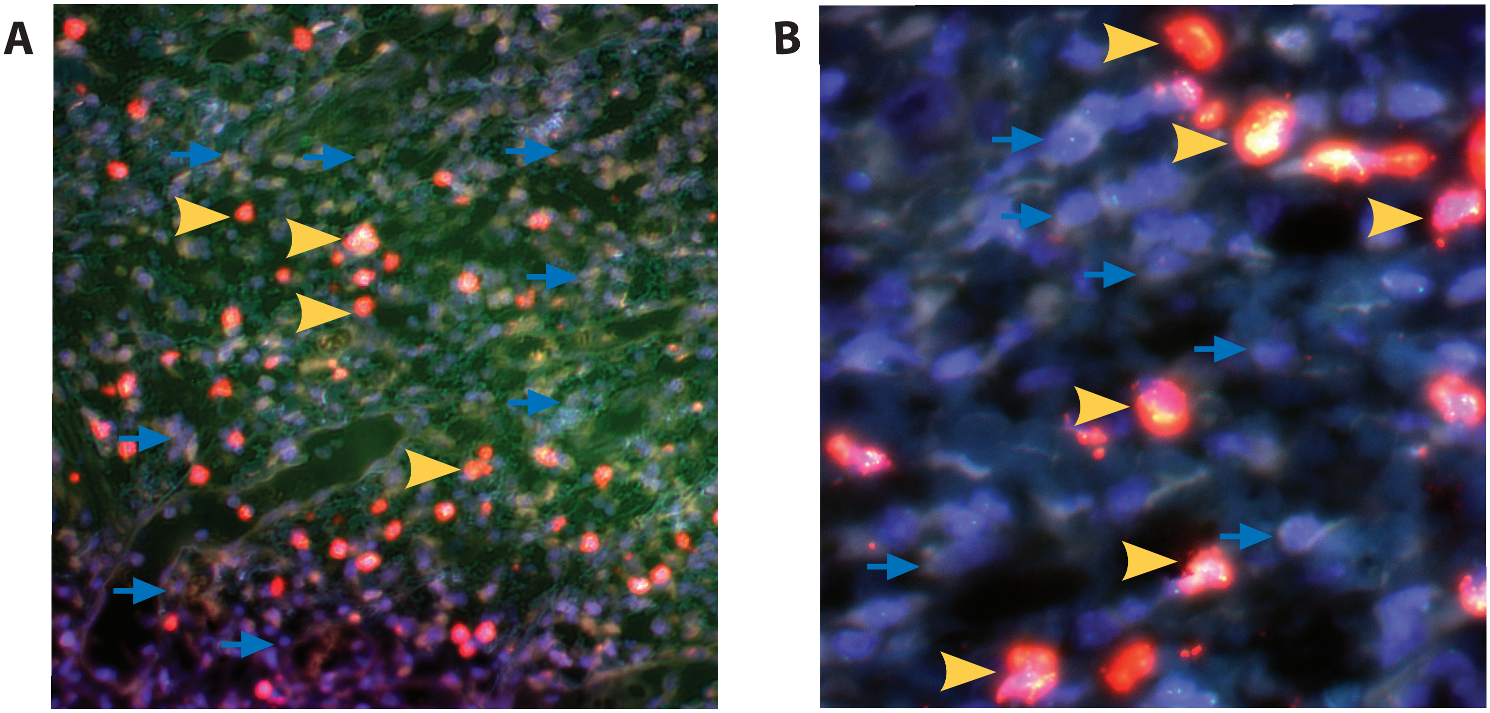

Supplementary Figure 2. FISH images demonstrated intratumoral heterogeneity with numerous clusters of OSCC cells with markedly high levels of EGFR-amplification (arrowheads), surrounded by OSCC cells with similar histologic appearance but no amplification (arrows), at 20x (A) and 400x (B) magnification.
