# Supplementary material for: Oral Squamous Cell Carcinoma in Young Patients Show Higher Rates of EGFR Amplification: Implications for Novel Personalized Therapy
Source: Front Oncol. 2021 Nov 29;11:750852. doi: 10.3389/fonc.2021.750852 (PMC8666981; doi:10.3389/fonc.2021.750852)
Supplement: Supplementary file 5 [file Image_5.pdf]

Supplementary figure 5

A

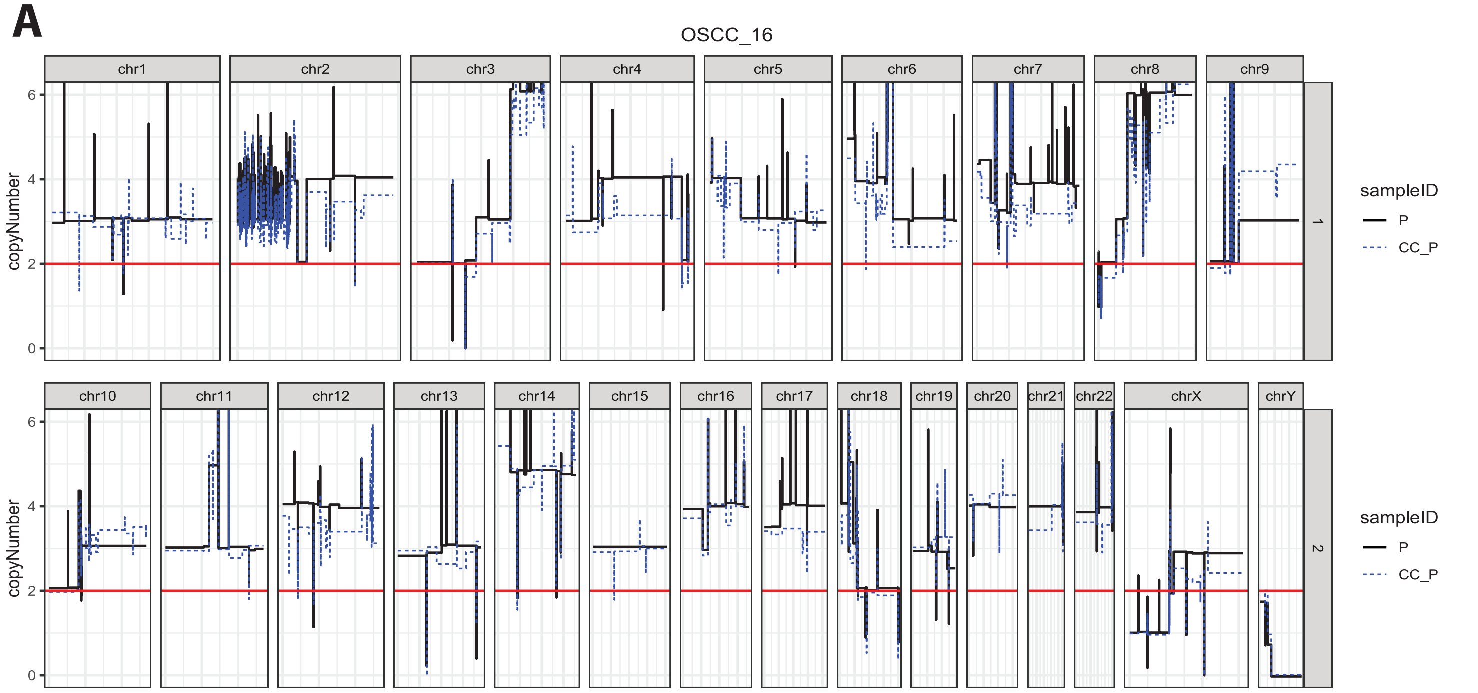

B

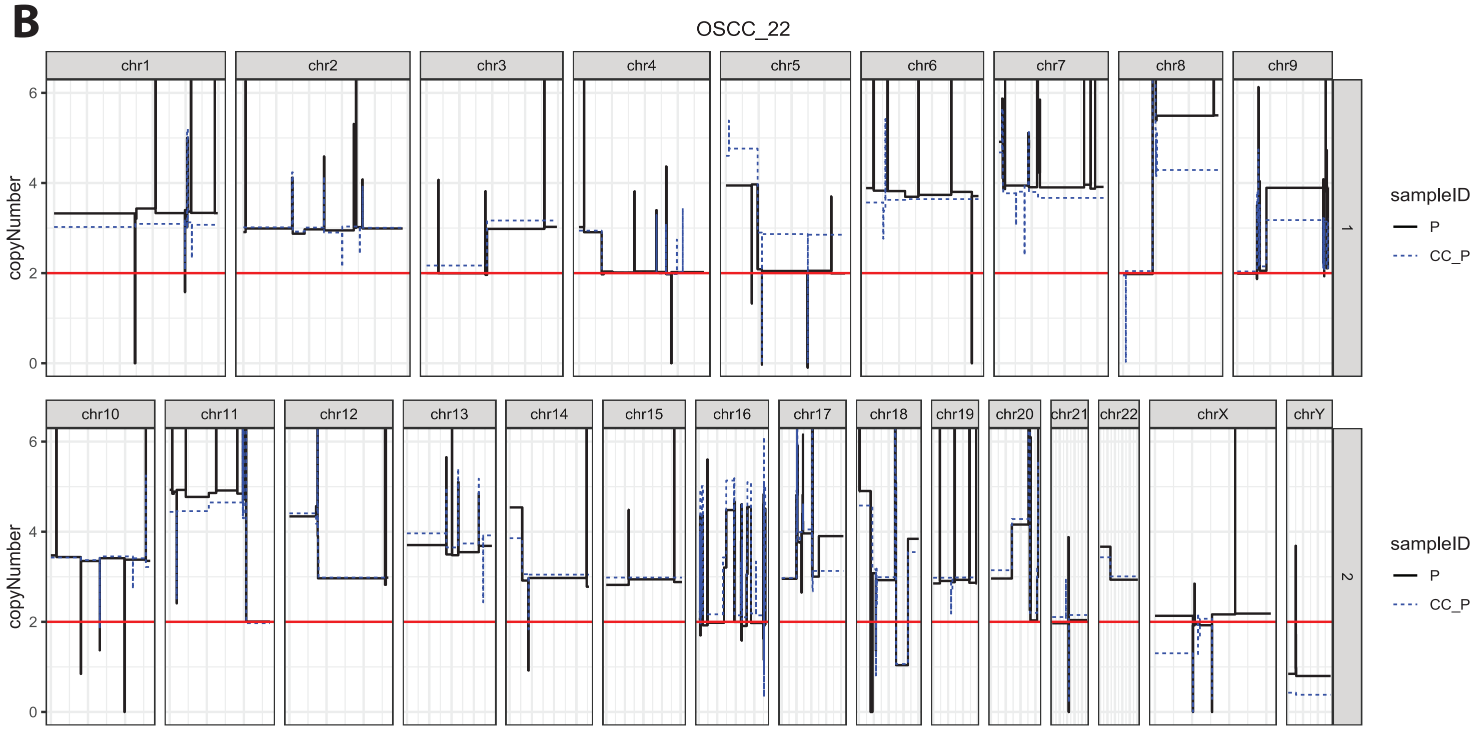

Supplementary Figure 5. Linearised copy number plots between matched patient tumour samples and PDCLs (A) with EGFR amplification and (B) without EGFR amplification.
